# Determinants of Delayed Breast Cancer Diagnosis: A Sample From the Mohammed VI Center for Cancer Treatment, Morocco

**DOI:** 10.7759/cureus.89191

**Published:** 2025-08-01

**Authors:** Sbabou Mohammed, Karima Bendahhou, Errahmani Mohamed-Yassir, Mouafik Abdelilah, Tahiri Jouti Nadia

**Affiliations:** 1 Laboratory of Cellular and Molecular Inflammatory, Degenerative and Oncologic Pathophysiology, Faculty of Medicine and Pharmacy, Hassan II University, Casablanca, MAR; 2 Department of Epidemiology and Public Health, Ibn Rochd University Hospital, Casablanca, MAR; 3 Department of Epidemiology and Public Health, Desbrest Institute of Epidemiology and Public Health, University of Montpellier, Institut National de la Santé et de la Recherche Médicale (INSERM), Montpellier, FRA; 4 Laboratory of Engineering and Applied Technologies, Faculty of Science and Technology, Sultan Moulay Slimane University, Beni Mellal, MAR

**Keywords:** breast cancer, delayed diagnosis, determinants, morocco, personal factors

## Abstract

Background: A delayed diagnosis of breast cancer (BC) is linked to more advanced stages of the disease and lower survival rates. This study aims to explore the association between personal factors, family and community interactions, awareness of BC and breast self-examination (BSE), and delays in BC diagnosis among patients at the Mohammed VI Oncology Center, Casablanca, Morocco.

Methods: We conducted a cross-sectional study at the Mohammed IV Oncology Center from January 2023 to July 2024. A questionnaire was administered to collect data on sociodemographic and clinical characteristics of participants, personal factors, and community and family interactions, as well as their knowledge about BC and BSE practices. BC stages I and IIa were categorized as "early diagnosis," while stages IIb, IIIa, IIIb, and IV were classified as "delayed diagnosis." A time interval of more than three months was considered delayed. The chi-square test or Fisher’s exact test was employed to assess the association between independent variables and outcomes. To examine the impact of the included factors on the interval of time (<3 months or >3 months), binary logistic regression analyses were conducted. Variables achieving statistical significance (p < 0.05) were subsequently included in the final multiple logistic regression models for each outcome.

Results: A total of 436 patients were interviewed, with a majority (65.1%) diagnosed at an advanced stage. The main factors impacting diagnosis interval included the individual’s residency in rural or semi-urban areas (OR: 2.01, 95% CI: 1.01-4.00), fear of death (OR: 0.97, 95% CI: 0.95-0.99), fear of total mastectomy (OR: 5.44, 95% CI: 1.39-21.32), distrust in the expertise of institutions (OR: 9.07, 95% CI: 1.52-53.88), and having never heard of BC (OR: 4.79, 95% CI: 1.49-15.41). Conversely, performing BSE (OR: 0.35, 95% CI: 0.12-1.01) was identified as a significant protective factor against delayed diagnosis.

Conclusion: It is crucial to focus on enhancing access to affordable, high-quality healthcare and to promote health literacy related to BC among the general public.

## Introduction

Breast cancer (BC) represents a major public health concern. As reported by the International Agency for Research on Cancer (IARC), it emerged as the predominant cancer diagnosis among women worldwide by 2022 [1]. In Morocco, analogous epidemiological patterns are observed, with BC accounting for 38.8% (n=12,756) of newly diagnosed female cancer cases [2]. Specifically, in Casablanca, a standardized incidence rate of 49.3 per 100,000 BC women was documented, based on the 2018-2021 incidence report from the Grand Casablanca Cancer Registry.

Early diagnosis and prompt initiation of therapeutic interventions demonstrate a significant association with improved survival rates [3,4]. Notwithstanding this, women in both developed and developing countries frequently encounter delays exceeding 90 days in their presentation and subsequent diagnosis [5-8]. Such delays critically compromise the effective management of BC. The Moroccan healthcare system, in particular, is confronted with comparable challenges for BC patients [9-11].

To address this public health issue, several studies have been conducted over time in various locations to identify its determinants. Key contributing factors identified in these studies encompass socioeconomic disparities, insufficient public awareness, and the apprehension of mortality linked to a BC diagnosis. Within the Moroccan context, empirical research specifically addressing personal determinants, familial and communal impacts, and the prevailing community knowledge, attitudes, and practices regarding BC remains notably limited.

Given the existing research gap and the urgent need to understand the factors influencing BC care-seeking behavior in Morocco, this study aims to investigate the association between personal factors, family and community interactions, awareness of BC and BSE, and delays in BC diagnosis among patients at the Mohamed VI Oncology Center, Casablanca, Morocco. The primary research question guiding this study was as follows: What are the key determinants affecting diagnosis intervals among BC patients in the Moroccan healthcare context? Specifically, we sought to address the following questions: How do personal factors impact the timing of BC diagnosis? What role do family and community interactions play in BC care-seeking behavior? To what extent does knowledge about BC and BSE diagnostic intervals?

We hypothesized that personal factors, such as age, education level, and socioeconomic status, would significantly influence the intervals for diagnosis. Additionally, we proposed that strong familial and community support would be associated with shorter delays in seeking care and that a higher level of knowledge about BC and BSE would correlate with earlier diagnosis.

This cross-sectional study was conducted among BC patients receiving care at the Mohamed VI Oncology Center, an integral component of Ibn Rochd University Hospital in Casablanca. This institution stands as a pivotal facility in the provision of oncological care within Morocco. A comprehensive understanding of perceptions and practices of the target population, alongside environmental influences, is anticipated to facilitate the identification of critical factors impacting BC presentation and diagnosis intervals, ultimately enhancing disease management strategies and improving patient outcomes.

## Materials and methods

Study design and setting 

A cross-sectional study was conducted to investigate the association between the personal factors, family and community interactions, knowledge levels regarding BC and breast self-examination (BSE), and the delayed diagnosis of BC patients. The study was conducted at the Mohamed VI Oncology Center, an integral part of the Ibn Rochd University Hospital in Casablanca, Morocco.

Participants

The study population comprised Moroccan female patients, aged 18 or older, who were newly diagnosed with BC (incident cases), and who provided informed consent. Participants were recruited from patients admitted to and followed up at the Mohammed VI Oncology Center of Ibn Rochd University Hospital in Casablanca during the study period. Recruitment was carried out using a convenience sampling strategy.

Data collection

Population recruitment occurred between January 2023 and July 2024. Data were collected through a structured questionnaire, along with relevant clinical information extracted from medical records. The questionnaire was developed following a narrative review of existing literature and was validated for content by clinical experts. It also underwent pilot testing with 20 participants. To minimize recall and information bias, several strategies were implemented during data collection. Data were obtained through structured in-person interviews conducted during patient consultations at the center, supplemented by information from medical records. To enhance accuracy in recall, we used temporal anchors, such as religious holidays and national events, to help patients remember specific dates and timeframes. The questionnaire consisted of two distinct modules. The first module gathered information on patient-related factors, including sociodemographic characteristics, clinical data, personal factors, and community and family interactions. The second module assessed participants' knowledge regarding BC and BSE and their practices.

The following variables were collected.

Sociodemographic and Clinical Variables

These are age, educational level, monthly household income, matrimonial status, health insurance, and type of health insurance (CNOPS: The National Fund for Social Security Organizations is a Moroccan institution). It is responsible for managing health insurance for the public sector and students. CNSS: The National Social Security Fund is a public institution in Morocco that constitutes a social security system mandatory for employees in industry, services, and liberal professions. Profession, residence area, and clinical staging (TNM) according to the American Joint Committee on Cancer (AMJCC).

Personal Perception

It refers to belief in the effectiveness of treatment, belief in the institution's expertise, and the fear (of diagnostic results, of total mastectomy, of problems with the partner, or of death).

Knowledge About BC, BSE, and Its Practice

This refers to knowledge about BC (heard about it, source of information, alarming signs, women at risk, awareness about BSE (recommended frequency, age, time), and its practice).

Community and Family Interaction

This refers to partners', children's, and family's perception of the disease, their willingness to consult, and their interactions and impacts.

Diagnosis Delay Definition

In this study, diagnostic delay was evaluated using two distinct methods, allowing for the evaluation of associated variables for each definition: the time interval and BC stage at diagnosis. Both definitions are widely recognized in the existing literature. In the stage-based delay, diagnosis was categorized as “late” when the cancer stage was classified as stage IIb or higher according to the AJCC criteria and “early” if classified as stage I or IIa. In the time interval delay, diagnostic delay was defined as a time interval exceeding 90 days between the onset of the first symptoms and the confirmed diagnosis of BC.

Data analysis

A descriptive analysis was conducted to characterize the study population, including measures of central tendency and dispersion for quantitative variables and frequencies for qualitative variables. Bivariate analysis was performed using the chi-square test or Fisher's exact test. All variables were subsequently included in a multivariate analysis model utilizing the stepwise backward method with multi-test corrections. Variables with p<0.5 were retained in the final model. The Hosmer and Lemeshow test was performed to assess the model fit. All statistical analyses were performed using Jamovi (version 2.3.28; https://www.jamovi.org).

Ethical consideration

Ethical approval for this study was obtained from the Ethics Committee for Biomedical Research of Rabat (CERB), under ethical clearance number 157/22. Participation in this study was entirely voluntary. Formal informed consent was obtained from all individuals who agreed to participate after receiving a comprehensive information sheet that provided a complete explanation of the purpose of the study. Participants were assured of anonymity, with no identifying information collected. All gathered data were maintained with strict confidentiality and utilized exclusively for research purposes.

## Results

Descriptive analysis

Descriptive Characteristics of the Study Population

 A total of 436 participants was included. Their mean age was 54.0 ± 12.0 years. Regarding the total diagnosis interval time, the median time was 30.0 days (interquartile range (IQR): 15.0-90.0 days). More than half were aged between 40 and 56 years old (57.8%) (n=252), illiterate (53.3%) (n=227), and reported living as a couple (59.8%) (n=261). The majority of participants were housewives (67.2%) (n=293) and had state-funded health insurance (76.3%) (n=319) (Table 1).

**Table 1:** Sociodemographic characteristics of participants CNSS: National Social Security Fund; CNOPS: National Social Security Fund for Public Sector Employees

Variables	n	%
Age		
>60	139	31.8
40-59	252	57.8
<40	45	10.3
Living as a couple		
No	173	39.6
Yes	261	59.8
Profession		
Housewife	293	67.2
Not employed	108	24.7
Employed	35	8.0
Education level		
None	227	53.3
Primary	102	23.9
Secondary or higher education	97	22.8
Residence area		
Rural or semi-urban	117	26.8
Urban	319	73.2
Monthly income (MAD)		
<4000	425	97.5
>4000	11	2.5
Health insurance		
No	23	5.3
Yes	412	94.5
Type of health insurance (n=418)		
State-funded health insurance	319	76.3
CNSS	78	18.7
CNOPS	14	3.3
Others	7	1.7

Description of Clinical Characteristics of Participants and Perceptions of Factors Contributing to Delayed Healthcare-Seeking Behavior

Approximately two-thirds of the participants were diagnosed at an advanced stage (65.1%) (n=284), despite the fact that the majority (80.9%) (n=309) sought medical consultation within three months of symptom onset. Participants were asked about their perceptions of some barriers that may have delayed their initial medical visit. While 57.5% (n=249) of the women expressed that they trust in the effectiveness of treatment, and 97.5% (n=421) trusted the professional competence of the healthcare institution, many reported significant emotional barriers. The most frequently cited fears included fear of diagnostic results (80.6%) (n=350), fear of total mastectomy (79.4%) (n=344), and fear of death (84.7%) (n=355). Regarding family support, the vast majority of participants reported full acceptance from both their partners (97.7%) (n=253) and their children (98.6%) (n=288) regarding the decision to seek diagnosis and treatment (see Table 2).

**Table 2:** Clinical characteristics of participants and their perceptions of factors contributing to delayed healthcare-seeking behavior

Variables	n	%
Stage at diagnosis		
Late	284	65.1
Early	152	34.9
Symptom-to-diagnosis interval		
>3 Months	73	19.1
<3 Months	309	80.9
Trust in the effectiveness of the treatment		
No	22	5.1
Yes, but it’s quite a heavy burden	162	37.4
Yes	249	57.5
Trust in the institution’s professional competence		
No	11	2.5
Yes	421	97.5
Concern over unfavorable test results		
Yes	350	80.6
No	84	19.4
Fear of full breast excision		
Yes	344	79.4
No	89	20.6
Fear of dying		
Yes	355	84.7
No	64	15.3
Concerns about potential issues with the partner		
Yes	65	23.3
No	213	76.6
Acceptance of medical care by partners (N=259)		
Yes	253	97.7
Accept with a condition (checked by a female doctor)	3	1.2
No	3	1.2
Acceptance of medical care by children (N=292)		
Unconditional acceptance	288	98.6
Accept with a condition (checked by a female doctor)	4	1.4
Family's perception of the disease		
Pessimism	53	12.4
Ignoring concerns related to the disease	32	7.5
Worry about financial costs	42	9.9
Realistic perception	299	70.2

Breast Self-Examination (BSE): Awareness and Practice

The majority of participants (94.0%) (n=410) indicated prior awareness of BSE, while slightly more than half (59%) (n=257) demonstrated knowledge of BC symptoms. The most commonly identified symptoms were “breast lump or axillary swelling” (62.6%) (n=161) and “change in the shape or size of the nipple” (16.7%) (n=43). Approximately one quarter (28%) (n=122) of participants reported knowing how to perform BSE; however, the vast majority were unaware of the recommended age to initiate BSE (99%) (n=434) and the appropriate frequency with which it should be practiced (96.8%) (n=421). With regard to actual practice, only 8.3% (n=36) of participants reported regularly performing BSE (see Table 3).

**Table 3:** Awareness and practice of breast self-examination among participants BC: Breast cancer; BSE: Breast cancer examination

Questions	n	%
Have you ever heard of breast cancer?		
No	25	5.7
Yes	410	94.0
Have you heard about the symptoms and warning signs of BC?		
Yes	257	59.0
No	175	40.1
Most recognized signs and symptoms (N=257)		
Breast lump or axillary swelling	161	62.6
Change in the shape/size of nipple	43	16.7
Rash/ulcer on the nipple/change in skin color	14	5.4
Inverted nipple	14	5.4
Breast heaviness	11	4.3
Dimpling of the breast skin	8	3.1
Abnormal discharge/blood from nipple	6	2.3
Have you heard of breast self-examination?		
No	313	71.8
Yes	122	28.2
From what age do you think breast cancer can appear in women?		
Any age	411	95.1
50 years old or more	21	4.9
At what age should women start BSE?		
No idea/wrong answer	434	99.8
Starting at the age of 20	1	0.2
How frequently should breast self-examination be done?		
No idea/wrong answer	421	96.8
Monthly	14	3.2
When is breast self-examination recommended?		
No idea	432	99.3
After the menstrual cycle	3	0.7
Where do you get your information from?		
Family discussions	171	41.7
Mass media	126	30.7
Social media	59	14.4
Discussions with friends/colleagues	43	10.5
During participation in awareness campaigns	5	1.2
Scientific articles/newspapers	3	0.7
Health professionals	3	0.7
Do you perform breast self-examination?		
No	399	91.5
Yes	36	8.3

Influence of Sociodemographic Factors on Diagnostic Delay

Among the variables analyzed, only place of residence and type of health insurance were significantly associated with diagnostic delay. More precisely, 23.6% of patients diagnosed within three months were from rural areas, compared to 43.8% among those diagnosed after three months (p < 0.001). Regarding health insurance, patients covered by the state-funded scheme accounted for 83.1% of those with a delay greater than three months versus 75.5% in the group with a shorter delay. CNSS coverage was reported in 9.9% and 20.5% of the delayed and non-delayed groups, respectively, while CNOPS beneficiaries represented 2.8% and 3.4% (p = 0.02) (see Table 4).

**Table 4:** Sociodemographic determinants of diagnostic delay CNSS: National Social Security Fund; CNOPS: National Social Security Fund for Public Sector Employees

Variables	>3 Months	<3 Months	p-value
Age			0.37
>60	30.4	30.1	
40-59	60.9	58.3	
<40	8.7	11.6	
Marital status			0.49
Not living as a couple	39.7	38.8	
Living as a couple	60.3	61.2	
Profession			0.35
Housewife	72.6	63.8	
Not employed	21.9	28.2	
Employed	5.5	8.1	
Education level			0.08
None	63.0	48.5	
Primary	19.2	25.9	
Secondary or higher education	17.8	25.6	
Residence area			<0.00
Rural or semi-urban	43.8	23.6	
Urban	56.2	76.4	
Monthly income (DH)			0.11
<4000	100.0	96.8	
>4000	0.0	3.2	
Health insurance			0.44
No	4.1	5.5	
Yes	95.9	94.5	
Type of health insurance (N=418)			0.02
State-funded health insurance	83.1	75.5	
CNSS	9.9	20.5	
CNOPS	2.8	3.4	
Others	4.2	0.7	

Fear of total mastectomy and death, along with family perceptions of the illness and children’s attitudes toward medical care, were significantly associated with diagnostic delay. Among participants diagnosed after more than three months, 89.1% reported a fear of total mastectomy, compared to 79.7% in those diagnosed earlier (p = 0.04). Interestingly, fear of death was more frequently reported among women with a shorter diagnostic delay (90.0% vs. 78.6%; p < 0.001). Regarding family influence, some participants indicated that their children accepted their decision to seek treatment only on the condition that the physician in charge was a woman. This was reported by 1.9% of women in the group with delayed diagnosis, compared to 0.5% in the group diagnosed earlier (p = 0.04) (see Table 5).

**Table 5:** Association between diagnostic delay and personal and familial perceptions of breast cancer

Questions	>3 Months	<3 Months	p-value
Trust in the effectiveness of the treatment			0.12
No	6.8	5.5	
Yes, but it’s quite a heavy burden	30.1	43.3	
Yes	63.0	51.1	
Trust in the institution’s professional competence			0.07
No	5.5	1.6	
Yes	94.5	98.4	
Concern over unfavourable test results			0.42
Yes	86.1	82.1	
No	13.9	17.9	
Fear of full breast excision			0.04
Yes	89.1	79.7	
No	10.9	20.3	
Fear of dying			0.008
Yes	78.6	90.0	
No	21.4	10.0	
Concerns about potential issues with the partner			0.11
Yes	15.2	26.1	
No	84.8	73.9	
Acceptance of medical care by partners			0.98
No	0.0	1.6	
Accept with a condition (checked by a female doctor)	0.0	1.6	
Yes	100.0	96.9	
Acceptance of medical care by children			0.04
Accept with a condition (checked by a female doctor)	1.9	0.5	
Unconditional acceptance	98.1	99.5	
Family's perception of the disease			0.0002
Pessimism	4.1	15.3	
Ignoring concerns related to disease	12.3	7.6	
Worry about financial costs	21.9	7.3	
Realistic perception	61.6	69.8	

Knowledge of BC and its symptoms, as well as the practice of BSE, were significantly associated with diagnostic delay. Specifically, 10.9% of women in the late-diagnosis group had never heard of BC, compared to 4.2% in the early-diagnosis group (p = 0.02). A similar pattern was observed regarding symptom awareness: 54.2% of women in the late-diagnosis group reported not knowing the symptoms of BC, versus 38.2% among those diagnosed earlier (p = 0.01) (see Table 6).

**Table 6:** Impact of breast cancer knowledge on diagnostic delay BC: Breast cancer

Questions	>3 Months	<3 Months	p-value
Have you ever heard of breast cancer?			0.02
No	10.9	4.2	
Yes	89.1	95.8	
Have you heard about the symptoms and warning signs of BC?			0.01
No	54.2	38.2	
Yes	45.8	61.8	
Have you heard of breast self-examination?			0.50
No	68.5	72.4	
Yes	31.5	27.6	
From what age do you think breast cancer can appear in women?			0.26
Any age	91,8	95,1	
50 years old or more	8,2	4,9	
At what age should women start BSE?			0.80
No idea/wrong answer	100.0	99.7	
starting at the age of 20	0.0	0.3	
How frequently should breast self-examination be done?			0.27
No idea/wrong answer	94.5	96.8	
Monthly	5.5	3.2	
When is breast self-examination recommended?			0.47
I don't know	98.6	99.4	
After the menstrual cycle	1.4	0.6	
Do you perform breast self-examination?			0.02
No	83.6	92.2	
Yes	16.4	7.8	

Multivariate analysis

The multiple regression model for the total diagnostic interval revealed several significant independent variables (see Table 7). Participants residing in rural or semi-urban areas demonstrated a significantly increased likelihood of experiencing a diagnostic delay exceeding three months (OR: 2.01, 95% CI: 1.01-4.00), indicating residence as a significant sociodemographic factor. Personal factors, such as a lack of belief in the expertise of institution (OR: 9.07, 95% CI: 1.52-53.88), fear of total mastectomy (OR: 5.44, 95% CI: 1.39-21.32), and fear of death (OR: 0.97, 95% CI: 0.95-0.99) were significantly associated with prolonged diagnostic intervals. Regarding knowledge about BC and BSE, being uninformed about BC (OR: 4.79, 95% CI: 1.49-15.41) negatively impacted the overall diagnostic timeframe. Conversely, the practice of BSE emerged as a protective factor against delayed presentation and diagnosis (OR: 0.35, 95% CI: 0.12-1.01).

**Table 7:** Independent factors associated with delay in the total diagnosis interval BC: breast cancer; BSE: Breast self-examination; CI: Confidence interval; OR: Odds ratio Statistical significance: p < 0.05

Variables	OR	95% IC	p-value
				Residence area: "rural or semi-urban area"	2.01	1.01-4.00	0.04
Lack of belief in the institution's expertise	9.07	1.52-53.88	0.01
The fear of total mastectomy	5.44	1.39-21.32	0.01
The fear of death	0.97	0.95-0.99	0.01
Heard about BC	4.79	1.49-15.41	0.009
Practice of BSE	0.35	0.12-1.01	0.05

The final model was statistically significant, explaining approximately 34.5% of the variance and demonstrating a good fit to the data (chi-square test: 3.274, p = 0.916).

## Discussion

Key finding

In this study, 65.4% of participants experienced delayed BC diagnosis (stage at diagnosis IIb or more). Factors significantly associated with a higher risk of delayed diagnosis included living in rural or semi-urban areas, having a fear of death or total mastectomy, a lack of belief in the institution's expertise, never having heard of BC, and not performing BSE.

In our study, the median time from the onset of their first symptoms to being diagnosed with BC was approximately 30 days (n=259). This finding is lower than that reported in a study of Moroccan women in Casablanca, conducted at both public and private institutions, where the median patient interval was about 35 days and 51.7% (n=300) of cases were diagnosed at a late stage [12]. It is also lower than the study of Aloulou et al. [13], which included Moroccan women in Marrakech; their study found a median total diagnostic interval of 8.47 months [13]. Additionally, research by Hannaoui et al. [14] reported a median total diagnostic interval of 157 days, with the median patient interval being around 90 days. In contrast, a study by Maghous et al. [10] noted a median patient interval of six months, with many diagnoses occurring at a late stage. Notably, a shorter patient interval was observed in the findings of a study conducted at the National Institute of Oncology in Rabat, which reported a median patient interval of 20 days, with 46% (n=92) classified as having a late diagnosis (stages III and IV) [9].

Living in rural or semi-urban areas significantly contributes to delays in the total diagnostic interval for patients. Women in these locations often need to travel long distances for diagnosis and to complete their care, which can result in high transportation costs. This finding is consistent with the work of Benbakhta et al. [9], who studied Moroccan women in Rabat and did research involving patients from four major oncology centers located in Marrakech, Casablanca, Rabat, and Fez [15]. These results also align with other studies documented in the literature [11,16,17].

Our study found that the fear of death and concerns about total mastectomy led to delays in presentation and diagnosis. Findings from different studies revealed that statement [6,11,13]. This perception is not only limited to BC but also extends to all types of cancer. The term "cancer" generally evokes dread and is seen as a life-threatening disease in both low-income and high-income countries, resulting in a range of negative emotions such as depression and anxiety among women [18]. For African women, the breast represents more than just a fashion statement or sexual organ; it is an integral part of their identity as wives and mothers [19]. Therefore, the loss of a breast can lead to a significant loss of identity and femininity. Moreover, some participants expressed the belief that BC is incurable [20].

In our study, the children demonstrated a positive perception of consulting for medical issues, which emerged as a protective factor against delayed presentation. This finding aligns with results from a multinational analysis on delays in the diagnosis and treatment of BC, where support from family and friends was associated with shorter intervals [21]. Similarly, it is reflected in the outcomes of a systematic review exploring the factors that affect delayed presentation and diagnosis of BC in women from developing Asian countries [22].

The lack of trust in institutional expertise has been identified as a barrier to early presentation in our study. These findings were highlighted in a systematic literature review conducted in Egypt, Nigeria, Ghana, Kenya, and Libya, which noted that a lack of trust and confidence in access to healthcare affects the time taken for presentation and diagnosis [23]. Similar results have been reported in other studies [24].

In our studied population, the level of knowledge regarding BC and BSE was qualified as very low. This aligns with a study conducted within a South African community, where women's knowledge about BC was also relatively low. The attitudes toward BC were negative, and the majority of women had never performed any BC diagnostic methods [25]. Other studies in the literature support these findings [18,26,27].

Additionally, the absence of BSE practice among women in our study (91.5%) (n=399) was identified as a significant factor affecting the overall diagnosis interval. Results from a multinational analysis that included participants from 12 countries, representing a wide range of socioeconomic, geographical, and cultural contexts, indicated a significant association between self-examination and a shorter patient interval [28]. These results are consistent with other studies in the literature [29].

Public health implications

The delayed diagnosis and presentation of BC is a common issue in developing countries. Identifying the factors contributing to this problem is essential for developing specific public policies and support strategies aimed at improving healthcare management for BC patients. Our findings, along with results from other studies involving Moroccan women in various cities, indicate that several factors indirectly influence cancer awareness and perceptions. This underscores the necessity of promoting health literacy to empower individuals to control modifiable social determinants of health. These determinants encompass personal characteristics and social resources essential for individuals and communities to access, understand, evaluate, and utilize information and services to make informed health decisions.

An effective health literacy system should incorporate Social and Behavior Change Communication (SBCC) as a core component of its framework. This process involves conducting formative research, behavioral analysis, communication planning, monitoring, and creating supportive environments that encourage positive behaviors among individuals and communities. The three core components of SBCC are communication, behavior change, and social change. In other words, successful programs or interventions should engage individuals and communities on multiple levels: behavioral (performance techniques), social (social support, interpersonal media), sensory (materials, media), and cognitive (problem-solving, information).

In a systematic review assessing the effectiveness of interventions addressing health literacy in cancer care, online interventions demonstrated good outcomes. These included platforms connecting patients with clinics and each other, videos providing illness-specific information, surveys, media, and discussion sessions. However, technology was shown to have limited effects when used alone, indicating that a variety of accessible approaches should be employed to enhance effectiveness [29].

Study strengths and limitations

This study presents several notable strengths, being the first of its kind to comprehensively investigate the multifaceted factors influencing diagnostic time intervals among Moroccan women. Its robust design incorporated a broad spectrum of 20 variables, encompassing clinical, sociodemographic, personal, family, and community interactions, alongside knowledge and practices related to BC and BSE. This holistic approach allowed for the identification of a wide range of barriers and facilitators to timely diagnosis and presentation. However, the study is not without limitations. The reliance on a convenience sampling strategy, while practical for patient recruitment within a specific clinical setting, inherently restricts the generalizability of our findings to the broader Moroccan female population. Future research employing probability sampling methods would significantly enhance external validity. While the 90-day threshold for diagnostic delay is well-established in international literature and was selected based on clinical evidence and contextual considerations for the Moroccan healthcare system, future studies could benefit from sensitivity analyses using alternative thresholds or continuous delay measures. Finally, the predictive potential of our models may be constrained due to the relatively insufficient sample size.

## Conclusions

This finding highlights several significant associated factors that delay timely BC diagnosis in Morocco, providing evidence for the development of targeted policies and interventions. Future healthcare policies should aim to reduce the median patient intervals from 30 days to 14 days within the next two years. Additionally, efforts should be made to improve breast cancer awareness among the general population and to implement standardized BSE education in primary healthcare settings, institutions, and universities. Looking ahead, integrating new technologies, developing culturally adapted patient navigation programs, and establishing multidisciplinary care pathways represent promising strategies for systematic improvement. These findings support a shift towards patient-centered care that addresses both structural healthcare barriers and community-level health literacy, ultimately leading to improved BC outcomes.

## References

[1] (2025). Absolute numbers, incidence, females, in 2022: continents. https://gco.iarc.fr/today/en/dataviz/bars?mode=cancer&sexes=2&key=total&group_populations=1&types=0.

[2] (2025). Absolute numbers, incidence, females, in 2022: Morocco. https://gco.iarc.fr/today/en/dataviz/pie?mode=cancer&sexes=2&populations=504.

[3] Parkin DM, Fernández LM (2006). Use of statistics to assess the global burden of breast cancer. Breast J.

[4] Kothari A, Fentiman IS (2003). Diagnostic delays in breast cancer and impact on survival. Int J Clin Pract.

[5] Ozmen V, Boylu S, Ok E (2015). Factors affecting breast cancer treatment delay in Turkey: a study from Turkish Federation of Breast Diseases Societies. Eur J Public Health.

[6] Landolsi A, Gahbiche S, Chaafii R (2010). [Reasons of diagnostic delay of breast cancer in Tunisian women (160 patients in the central region of Tunisia)]. Tunis Med.

[7] Ermiah E, Abdalla F, Buhmeida A, Larbesh E, Pyrhönen S, Collan Y (2012). Diagnosis delay in Libyan female breast cancer. BMC Res Notes.

[8] Ukwenya AY, Yusufu LM, Nmadu PT, Garba ES, Ahmed A (2008). Delayed treatment of symptomatic breast cancer: the experience from Kaduna, Nigeria. S Afr J Surg.

[9] Benbakhta B, Tazi M, Benjaafar N, Khattabi A, Maaroufi A (2015). [Determinants of patient and health system delays for women with breast cancer in Morocco, 2013]. Rev Epidemiol Sante Publique.

[10] Maghous A, Rais F, Ahid S (2016). Factors influencing diagnosis delay of advanced breast cancer in Moroccan women. BMC Cancer.

[11] Soliman AA, Khouchani M, Renne EP (2019). Sociocultural barriers related to late-stage presentation of breast cancer in Morocco. J Cancer Educ.

[12] Zahfir H, Zoukal S, Hassoune S, Nani S (2022). Factors related to delayed diagnosis and treatment of breast cancer among Moroccan women in Casablanca. Indian J Gynecol Oncol.

[13] Aloulou S, El Mahfoudi A, El Omrani A, Khouchani M (2015). [Factors related to late diagnosis of breast cancer: experience of CHU Mohammed VI Marrakech]. Pan Afr Med J.

[14] Hannaoui M, Bourkeddi S, Lamarti A, Madrane M, Sfendla A (2022). Health education and health literacy correlation: the case of the factors affecting breast cancer screening. Education Excellence and Innovation Management: A 2025 Vision to Sustain Economic Development during Global Challenges.

[15] Ellis GK, Manda A, Topazian H (2021). Feasibility of upfront mobile money transfers for transportation reimbursement to promote retention among patients receiving lymphoma treatment in Malawi. Int Health.

[16] Yau TK, Choi CW, Ng E, Yeung R, Soong IS, Lee AW (2010). Delayed presentation of symptomatic breast cancers in Hong Kong: experience in a public cancer centre. Hong Kong Med J.

[17] Sauvaget C, Boutayeb S, Bendahhou K (2023). The journey of cancer patients and the quest to equity: findings from Morocco. Public Health.

[18] Petrova D, Garrido D, Špacírová Z (2023). Duration of the patient interval in breast cancer and factors associated with longer delays in low-and middle-income countries: a systematic review with meta-analysis. Psychooncology.

[19] Merriman A (2010). Emerging breast cancer epidemic: impact on palliative care. Breast Cancer Res.

[20] Andersen RS, Vedsted P, Olesen F, Bro F, Søndergaard J (2009). Patient delay in cancer studies: a discussion of methods and measures. BMC Health Serv Res.

[21] Jassem J, Ozmen V, Bacanu F (2014). Delays in diagnosis and treatment of breast cancer: a multinational analysis. Eur J Public Health.

[22] Sobri FB, Bachtiar A, Panigoro SS (2021). Factors affecting delayed presentation and diagnosis of breast cancer in Asian developing countries women: a systematic review. Asian Pac J Cancer Prev.

[23] Donkor A, Lathlean J, Wiafe SA (2015). Factors contributing to late presentation of breast cancer in Africa: a systematic literature review. Arch Med.

[24] Otieno ES, Micheni JN, Kimende SK, Mutai KK (2010). Delayed presentation of breast cancer patients. East Afr Med J.

[25] Ramathuba DU, Ratshirumbi CT, Mashamba TM (2015). Knowledge, attitudes and practices toward breast cancer screening in a rural South African community. Curationis.

[26] Baig M, Sohail I, Altaf HN, Altaf OS (2019). Factors influencing delayed presentation of breast cancer at a tertiary care hospital in Pakistan. Cancer Rep (Hoboken).

[27] Akuoko CP, Armah E, Sarpong T, Quansah DY, Amankwaa I, Boateng D (2017). Barriers to early presentation and diagnosis of breast cancer among African women living in sub-Saharan Africa. PLoS One.

[28] Jeitani C, Van den Broucke S, Leemans C (2025). Interventions addressing health literacy in cancer care: a systematic review of reviews. Int J Environ Res Public Health.

[29] Harirchi I, Ghaemmaghami F, Karbakhsh M, Moghimi R, Mazaherie H (2005). Patient delay in women presenting with advanced breast cancer: an Iranian study. Public Health.

